# Medium and long‐term efficacy of psychoeducational family intervention for bipolar I disorder: Results from a real‐world, multicentric study

**DOI:** 10.1111/bdi.13182

**Published:** 2022-02-09

**Authors:** Mario Luciano, Gaia Sampogna, Valeria Del Vecchio, Vincenzo Giallonardo, Arcangelo Di Cerbo, Carmela Palummo, Claudio Malangone, Debora Lampis, Franco Veltro, Francesco Bardicchia, Giusy Ciampini, Emanuele Orlandi, Annamaria Moroni, Silvia Biondi, Massimiliano Piselli, Giulia Menculini, Giuseppe Nicolò, Enrico Pompili, Giuseppe Carrà, Andrea Fiorillo

**Affiliations:** ^1^ Department of Psychiatry University of Campania “Luigi Vanvitelli” Naples Italy; ^2^ Mental Health Centre of Ravello Ravello Italy; ^3^ Mental Health Centre of Lanusei Lanusei Italy; ^4^ Mental Health Department of Campobasso Campobasso Italy; ^5^ Mental Health Centre of Grosseto Grosseto Italy; ^6^ Mental Health Centre of Lanciano Lanciano Italy; ^7^ Mental Health Centre of Sassuolo Sassuolo Italy; ^8^ 9338 Department of Psychiatry Niguarda Hospital Milan Italy; ^9^ Mental Health Centre of Montecatini Montecatini Italy; ^10^ Mental Health Centre of Foligno Foligno Italy; ^11^ 9309 Department of Psychiatry University of Perugia Perugia Italy; ^12^ Mental Health Centre of Colleferro Colleferro Italy; ^13^ Department of Medicine and Surgery University of Milano Bicocca Monza Italy

**Keywords:** bipolar I disorder, family, long‐term efficacy, psychoeducation, real‐world

## Abstract

**Objectives:**

This study aims to explore the long‐term efficacy of a psychoeducational family intervention (PFI) in bipolar I disorder at one and five years post‐intervention in terms of improvement of: (1) patients’ symptoms and global functioning and (2) relatives’ objective and subjective burden and coping strategies.

**Methods:**

This is a multicentre, real‐world, controlled, outpatient trial. Recruited patients and key‐relatives were consecutively allocated to the experimental intervention or treatment as usual. Patients were assessed at baseline, and after one and five years.

**Results:**

One hundred and thirty‐seventh number families have been recruited; 70 have been allocated to the experimental intervention, and 67 have been allocated to the control group. We observed an increasing positive effect of the PFI on patients’ clinical status, global functioning and objective and subjective burden after one year. We also found a reduction in the levels of relatives’ objective and subjective burden and a significant improvement in the levels of perceived professional support and of coping strategies. The efficacy of PFI on patients’ clinical status was maintained at five years from the end of the intervention, in terms of relapses, hospitalizations and suicide attempts.

**Conclusions:**

The study showed that the provision of PFI in real‐world settings is associated with a significant improvement of patients’ and relatives’ mental health and psychosocial functioning in the long term. We found that the clinical efficacy of the intervention, in terms of reduction of patients’ relapses, hospitalization and suicide attempts, persists after 5 years. It is advisable that PFI is provided to patients with BD I in routine practice.

## 1 INTRODUCTION

Bipolar disorder (BD) is a chronic psychiatric illness, characterized by an alternance of depressive and manic or hypomanic episodes. In addition to mood instability, BD is associated with significant functional impairment, low quality of life, and high suicide rate.^1^, ^2^ Up to 50% of individuals with bipolar I disorder do not recover from severe manic episodes within one year, and only about 25% return to their previous level of functioning.^3^ While psychotropic medications remain the mainstream treatment for bipolar disorder,^4^ pharmacotherapy alone allows remission only to a minority of patients.^5^

BD is associated with severe levels of burden on family members and on the society at large due to recurrent mood episodes, frequent hospitalizations, and loss of productivity.^3^ In particular, several studies found high levels of burden, expressed emotions,^6^, ^7^ dysfunctional coping strategies and significant morbidity^8^ in the majority of relatives. In fact, taking care of a person with BD is very a demanding task characterized by a sense of powerlessness, hopelessness, and inability to positively cope with the situation.^9^

High levels of family burden are associated with poor patients’ outcome, significant morbidity and mortality,^10^ poor treatment adherence and frequent relapses and hospitalizations.^3^ Moreover, patients living in families with high levels of burden have a reduced social functioning,^6^ a poor quality of life^11^ and present residual symptoms more frequently than patients living in families with low levels of family burden.^12^

Moreover, high levels of family burden are associated also with significant distress in relatives, who frequently show sleep disturbances, mild mental disturbances, depressive symptoms, psychosocial impairment, emotional exhaustion and a high utilization of mental and physical health care services.^10^

Despite the evidence of a massive involvement of relatives in the health care of patients with BD, family supportive interventions are provided only rarely. In particular, psychoeducational family interventions (PFI) are recommended by the most updated treatment guidelines for bipolar disorder, as adjunctive intervention to pharmacological treatment^13^, ^14^ for increasing patients’ and relatives’ knowledge about the illness, improving patients’ adherence to treatment, and reducing hospitalizations and recurrences.^15^ However, while most of available studies have explored the short‐term efficacy of these interventions, only a few studies have explored the medium and long‐term impact of PFI on clinical, social and family outcome of patients with bipolar I disorder. Moreover, most studies have been carried out in tertiary settings, with strict selection criteria and rigorous methodologies,^6^ being not generalizable to the real life of patients with BD and their family members.

This study, funded by the Italian Ministry of Health and coordinated by Department of Psychiatry of the University of Campania “L. Vanvitelli”, has been carried out in 11 randomly selected Italian mental health centres. The primary aim of our study was to explore the efficacy of the Falloon psychoeducational intervention^16^ in patients with bipolar I disorder and relatives as add‐on therapy to treatment as usual (TAU) compared to TAU alone in terms of: (1) improvement of patients’ global psychopathological status and global functioning; (2) reduction of relatives’ objective and subjective burden and improvement of coping strategies. Secondary analyses allowed us to evaluate the medium and long‐term efficacy of this intervention. In this paper we report the results of our study at 1 and 5 years after the intervention.

## 2 MATERIALS AND METHODS

This is a multicentric real‐world controlled study, which included the following phases: (1) randomly selection of 11 Italian mental health centres, stratified by geographical area and population density; (2) development of educational materials and selection of assessment instruments; (3) training of at least 2 mental health professionals in each participating centre on PFI and on assessment tools; (4) recruitment of at least 16 families of patients with bipolar I disorder in each centre.

Patients referring to the outpatient units of participating mental health centres have been invited to participate if they were: (a) aged between 18–65 years; (b) in charge to the local mental health centre for at least 6 months, with at least one access per month; (c) experiencing an affective episode in the previous three years; (d) living with at least one adult relative aged 18–70 years; (e) able to provide written informed consent. Patients who were not clinically stable at recruitment and those suffering from a severe and disabling chronic physical condition requiring intensive medical care were excluded.

All patients who agreed to participate were asked permission to contact and involve their key‐relative(s). For each patient, one or more key‐relative could be recruited. Key‐relatives were defined as those spending the highest number of hours in contact with the patient during the last year.

Patients and their relatives were consecutively allocated to receive the experimental intervention or the control group. Patients from both groups continued to receive the treatment usually provided in their centre (TAU), which included regular outpatient psychiatric assessment, pharmacological treatment and management of medications’ side effects. All patients received an adequate pharmacological treatment according to the NICE guidelines^6^ for the whole duration of the study. Patients who refused to take medications were excluded. More information on the study methodology is reported elsewhere.^6^ The study has been carried out in compliance with the ethical principles of the Declaration of Helsinki and has been approved by the Ethical Committee of the University of Campania “L. Vanvitelli (number: 9556/2009).

### 2.1 Training of mental health professionals

In each participating center, two mental health professionals (one of them being a psychiatrist) received a training course on the intervention and on the study protocol. The training consisted of three‐monthly sessions, each lasting two and half days (20 h per session). Five supervision meetings lasting one and half days were provided in order to support mental health professionals during the study period. An additional training course on the use of assessment tools and to test participants’ inter‐rater reliability has been carried out.

### 2.2 Assessment instruments

Sociodemographic and clinical characteristics of patients and relatives have been recorded through ad‐hoc schedules. Recorded information included diagnosis, illness duration, age at onset, number of affective episodes and of previous hospitalizations, age at first hospitalization, number of suicide attempts, age, gender, educational level, occupational status, pharmacological and psychosocial interventions.

The Brief Psychiatric Rating Scale (BPRS),^17^ a semi‐structured interview consisting of 24‐items, scoring from 1 (none) to 7 (very severe), grouped in four subscales: positive symptoms, negative symptoms, depressive symptoms and manic‐hostility symptoms, has been used to assess patients’ clinical status.

The Disability Assessment Schedule (DAS),^18^ which includes 11 areas (“self‐care”, “participation in daily activities”, “slowness”, “social withdrawal”, “family participation”, “affective and marital role”, “parental role”, “social contacts”, “occupational role”, “interest and information”, and “behavior in emergency situations”), has been used to assess patients’ social and personal functioning. A total score assessing patients’ overall general functioning is included at the end of the interview. Higher scores indicate a worse social functioning; each area of functioning ranges from 1 (excellent functioning) to 6 (very severe dysfunction).

The Personal Problems’ Questionnaire (PPQ), a self‐reported questionnaire including 34 items, grouped in 7 subscales (subjective and objective burden, practical and affective support, social and professional help, social network) has been used to assess patients’ burden of illness. Each item scores from 1 (“never”) to 4 (“always”)^19^ The same questionnaire, relatives’ version (Family Problem Questionnaire – FPQ), has been used to assess relatives’ objective and subjective burden.^20^

The Social Network Questionnaire (SNQ),^19^ a self‐administered questionnaire including 15 items grouped in 4 subscales (practical support, affective support, social and professional help, and help in emergency) has been used to assess patients’ and relatives’ social network. Items range from 1 (“never”) to 4 (“always”).

The Family Coping Questionnaire (FCQ),^19^ a self‐administered questionnaire consisting of 34 items, has been used to assess relatives’ coping strategies. Items are rated on a 4‐level scale, from 1 (“never”) to 4 (“always”), grouped into 11 subscales (seeking for information on patient's illness, positive communication toward the patient, relatives’ maintenance of social interests, patient's involvement in social activities, talking with friends about the patient's condition, coercion, avoidance, resignation, use of alcohol and drugs, and collusion).

Adherence to pharmacological treatment was mandatory to be included in the study. Pharmacological treatment regimen was considered adequate if at least one mood stabilizer or one atypical antipsychotic drug was prescribed, in accordance with the NICE guidelines for the management of bipolar disorder.^21^

Patients and relatives were assessed at baseline (T0), at the end of the intervention (T1), and after one year from the end of the intervention (T2). After five years (T3), information on the following course indicators were collected: number of relapses requiring a significant modification of the pharmacological treatment, number of hospitalizations and total length of hospitalizations, suicide attempts and number of suicide attempts. In the present paper T2 and T3 data are reported. T1 data have been reported elsewhere.^6^, ^8^

### 2.3 Inter‐rater reliability

Cohen's kappa coefficient for BPRS was between 1.0 and 0.90 for 43% of items, between 0.89 and 0.70 for 29%, and between 0.69 and 0.50 for the remaining 28% of items. Cohen kappa coefficient for DAS was between 1 and 0.90 for 39% of items, between 0.89 and 0.70 for 16%, and between 0.69 and 0.50 for the remaining 45% of items.

### 2.4 Description of the experimental intervention

The experimental intervention is based on the psychoeducational family intervention model developed by Falloon^16^ for patients with schizophrenia and their relatives. The Falloon model has been adapted to bipolar disorder by our research group to be used in Italian non‐tertiary settings, taking into account recent changes occurred in families’ composition and structure.^22^ The approach has been adapted to BD according to the following methodology: (1) analysis of scientific literature, handbooks and manuals on bipolar disorder^23^, ^24^, ^25^, ^26^; (2) focus groups with relevant stakeholders (researchers, expert clinicians, users and carers) in order to identify the most important components to be included in the intervention; (3) development or adaptation of the following sessions: (1) individual and family assessment; (2) information on clinical and course characteristics of BD, its treatment, early warning signs, management of suicidal behaviors; (3) communication skills; (4) problem solving skills.

Sessions are provided every 10 days (three times a month) for 4 to 6 months (about 12–18 sessions in total). Each session lasts about 90 min. Site and frequency of sessions are adapted to families’ needs and mental health professionals’ duties and workloads. Leaflets and other written materials are usually given to family members, whenever relevant.

### 2.5 Statistical analyses

Differences between patients’ and relatives’ socio‐demographic characteristics from the experimental and the control groups have been tested using χ^2^ or t‐test for independent samples, as appropriate. Differences at T0 and T2 samples, and at T0 and T3, with respect to patients’ and relatives’ socio‐demographic characteristics have been explored with χ^2^ or t‐test for independent samples, as appropriate. The impact of the intervention on patients’ social and clinical variables, as well as on relatives’ burden and coping strategies after one year in the two groups, has explored by the Student t‐test for paired samples. Linear regression models have been used to test the impact of socio‐demographic and clinical characteristics on patients’ clinical status and global functioning at one year. Logistic regression analyses have been carried out to test the efficacy of the experimental intervention on course indicators at five years. All clinical and socio‐demographic variables that were significantly different at the relevant univariate analyses, as well as other potential explanatory variables identified from the literature (i.e., age, gender, years of education, BPRS subscores at baseline, employment and number of relatives per each patient), have been included in the regression model. The T0 BPRS subscales’ scores have been used as independent variables in all regression analyses in order to correct multivariate models for the baseline symptom levels. Data analysis has been carried out using SPSS Statistical software, Version 18.0, with a significance level of *p *< 0.05.

## 3 RESULTS

One of the 11 centres did not provide the intervention and was excluded from the study. 143 families of patients with BD from the remaining 10 centres have been invited to participate. Of these, 137 agreed to participate and have been randomly allocated to the experimental (70 patients and 85 relatives) or the control group (67 patients and 70 relatives). Fourteen families dropped‐out during the first six month of the study (10 families from the experimental group and 4 from the control group), due to logistic difficulties in attending the sessions, lack of interest, onset of a severe physical disorder in one family member, illness exacerbation in the patient. The retention rate was 93% in the experimental group and 94% in the control group at T1, with a study sample of 123 families (60 patients and 72 relatives in the experimental group and 63 patients and 67 relatives in the control group). There have been no dropouts at T2. After 5 years (T3), a total of 23 patients (11 from the experimental and 13 from the control group) have not been reassessed due to patients’ death (1 for suicide and 4 for physical illnesses), onset of a severe Alzheimer disease (*N* = 1), or patients not being in charge anymore to the participating mental health centre (*N* = 11). Six patients were excluded from the analyses because they refused to take medications (two patients from the experimental and four from the control group). The retention rate at T3 was 70% in the experimental group and 75% in the control group, with a final study sample of 99 patients (49 in the experimental and 50 in the control group). There were no statistically significant differences at T0 between patients and relatives who completed the T3 assessments and those who dropped‐outs in terms of sociodemographic and clinical characteristics.

### 3.1 Patients’ socio‐demographic and clinical characteristics

The socio‐demographic characteristics of the global sample and of the two groups are reported in Table 1. At T0 there were no statistically significant differences between the two groups, with the exception of time in charge to the local mental health centre (74.9 ± 70.6 months in the experimental group vs. 103.2 ± 73.1 in the control group, *p *< 0.05) (Table 1).

**TABLE 1:** Socio‐demographic features of the patients at T0

	Total sample (*N* = 137)	Experimental group (*N* = 70)	Control group (*N* = 67)	*p*
Gender, F % (*n*)	62.7 (86)	60.3 (42)	65.7 (44)	NS
Age, M (sd)	47 (±11.1)	46.3 (10.0)	48.3 (±12.1)	NS
Marital status, married, yes, % (*N*)	60.3 (82)	64.3 (45)	56.1 (37)	NS
Level of education, % (*N*)				NS
Primary school degree	47.1 (64)	41.4 (29)	53 (35)	
High school degree	44.5 (61)	47.1 (33)	39.4(28)	
University degree	9.6 (13)	11.4 (8)	7.6 (5)	
Employed, Yes, % (*N*)	38.3 (55)	42.5 (31)	34.3 (23)	NS
Number of family members, M (sd)	3.3 (±1.1)	3.4 (±1.0)	3.2 (±1.1)	NS
Times in charge to MHC, months, M (sd)	88 (±72.8)	74.2 (±70.3)	103.3 (±73.7)	<.05
Duration of illness, years, M (sd)	14.6 (±9.5)	13.7 (±9.3)	15.8 (±9.7)	NS
No. voluntarily admission from onset of the disorder, M (sd)	2.7 (±3.6)	2.6 (±3.8)	2.9 (±3.4)	NS
No. voluntarily admission during the last year, M (sd)	0.4 (±0.7)	0.4 (±0.6)	0.5 (±0.8)	NS
No. involuntarily admission from onset of the disorder, M (sd)	1 (±3.1)	0.7 (±1.8)	1.4 (±4.1)	NS
Involuntarily admission during the last year; yes % (*N*)	7.1 (10)	5.5 (4)	9 (6)	NS
Suicide attempts, yes %(*N*)	23.4 (32)	23.6 (17)	21.7 (15)	NS
BPRS‐ positive symptoms; M (sd)	1.2 (±0.4)	1.2 (±0.3)	1.3(±0.4)	NS
BPRS‐ negative symptoms; M (sd)	1.4 (±0.5)	1.3 (±0.5)	1.5(±0.5)	NS
BPRS‐manic symptoms; M (sd)	1.3 (±0.5)	1.3 (±0.5)	1.3(±0.5)	NS
BPRS‐ depressive symptoms; M (sd)	2 (±0.7)	2.0 (±0.7)	2.0(±0.8)	NS
DAS‐ global score; M (sd)	2.9 (±0.9)	2.9 (±0.9)	2.9(±1.0)	NS

### 3.2 Relatives’ socio‐demographic characteristics

Relatives’ socio‐demographic characteristics are reported in Table 2. At T0 there were no statistically significant differences between treated relatives and those included in the control group.

**TABLE 2:** Socio‐demographic characteristics of relatives at T0

	Total sample N=155	Experimental group N=85	Control group N=70	*p*
Gender, F % (*N*)	58.7 (91)	58.3 (49)	60.0 (42)	NS
Age, M (sd)	51.7 (±13.5)	51.2 (13.7)	52.8 (13.6)	NS
Marital status, married, yes, % (*N*)	67.7 (105)	66.7 (56)	70 (49)	NS
Level of education, % (*N*)				NS
Primary school degree	54.4 (84)	53.6 (45)	55.7 (39)	
High school degree	38.6 (61)	36.9 (33)	40.0 (28)	
University degree	7 (11)	9.5 (8)	4.3 (3)	
Employed, Yes, % (*N*)	49.0 (76)	53.6 (45)	44.3 (31)	NS
Type of family member, % (*N*)				NS
Parent	28.7 (45)	25.3 (21)	32.9 (24)	
Spouse	48.4 (76)	51.8 (45)	44.3 (31)	
Son	14 (22)	12.0 (11)	15.5 (11)	
Sibling	7 (11)	9.6 (8)	4.3 (3)	
Other	1.9 (3)	1.2 (1)	2.9 (2)	
Years of cohabiting with patient, M (sd)	25.8 (±12.1)	24.4 (±12.2)	27.6 (±11.8)	NS
Hours daily spend with patient, M (sd)	6.8 (±3.6)	6.5 (±3.3)	7.2 (±4)	NS

### 3.3 Efficacy of psychoeducational family intervention on patients’ clinical status, global functioning and personal burden at one year

We found an increasing positive effect of the intervention on clinical status and global functioning was found in the experimental group. In particular, we observed a significant reduction at the BPRS positive (*p *< 0.05), negative (*p *< 0.01) and depression/anxiety symptoms’ subscales (*p *< 0.001), as well as a significant improvement at the DAS global score (*p *< 0.01) (Table 3). Moreover, we also found a significant improvement in patients’ objective (*p *< 0.001) and subjective burden (*p*<.001). There were no significant changes in the control group with respect to patients’ clinical status, social functioning and personal burden (Table 3).

**TABLE 3:** One‐year efficacy of the experimental intervention

	Patients (*N *= 123)						Relatives (*N *= 155)					
	Experimental intervention (*N *= 60)			Control group (*N *= 60)			Experimental intervention (*N *= 85)			Control group (*N *= 70)		
	T0 Mean (sd)	T2 Mean (sd)	*t* Student	T0 Mean (sd)	T2 Mean (sd)	*t* Student	T0 Mean (sd)	T2 Mean (sd)	*t* Student	T0 Mean (sd)	T2 Mean (sd)	*t* Student
DAS‐global score	2.9 (1.0)	2.5 (0.8)	3.0**	3.0 (1.0)	3.0 (1.0)	−0.293	NA	NA	NA	NA	NA	NA
Objective burden	2.1 (0.9)	1.7 (0.6)	3.8***	2.0 (0.8)	1.9 (0.8)	1.2	1.6 (0.7)	1.4 (0.4)	3.5**	1.6 (0.7)	1.6 (0.7)	0.5
Subjective burden	2.3 (0.8)	1.9 (0.6)	4.7***	2.2 (0.8)	2.1 (0.7)	2.2	2.0 (0.6)	1.6 (0.5)	5.6***	1.9 (0.6)	1.9 (0.7)	−1.0
Social contacts	2.2 (0.6)	2.3 (0.5)	−0.5	2.2 (0.5)	2.2 (0.5)	−1.1	2.2 (0.4)	2.3 (0.4)	−1.2	2.2 (0.4)	2.2 (0.4)	0.3
Practical support	2.8 (0.7)	2.8 (0.6)	−0.4	2.8 (0.7)	2.9 (0.6)	−1.4	2.4 (0.7)	2.6 (0.7)	−1.6	2.4 (0.7)	2.6 (0.6)	−2.2*
Affective support	2.4 (0.4)	2.4 (0.4)	−0.7	2.4 (0.4)	2.4 (0.4)	0.6	2.5 (0.6)	2.6 (0.6)	−1.6	2.4 (0.6)	2.5 (0.7)	−1.0
Professional help	2.8 (0.4)	2.8 (0.5)	0.6	2.8 (0.5)	2.8 (0.4)	0.1	2.9 (0.6)	3.3 (0.4)	−5.9***	2.9 (0.7)	3 (0.7)	−0.3
Help in emergencies	2.7 (0.5)	2.8 (0.4)	−1.6	2.6 (0.4)	2.6 (0.4)	−0.1	2.5 (0.3)	2.6 (0.3)	−1.7	2.5 (0.3)	2.5 (0.3)	−0.3
BPRS‐positive symptoms	1.2 (0.4)	1.1 (0.2)	2.7*	1.3 (0.4)	1.4 (0.6)	−1.5	NA	NA	NA	NA	NA	NA
BPRS‐negative symptoms	1.3 (0.5)	1.2 (0.4)	3.6**	1.4 (0.5)	1.4 (0.6)	−0.0	NA	NA	NA	NA	NA	NA
BPRS‐depression/anxiety symptoms	2.0 (0.7)	1.6 (0.7)	5.0***	2.1 (0.8)	1.9 (0.8)	1.5	NA	NA	NA	NA	NA	NA
BPRS‐manic/hostility symptoms	1.3 (0.5)	1.3 (0.6)	0.6	1.3 (0.5)	1.4 (0.8)	−1.128	NA	NA	NA	NA	NA	NA

Abbreviations: BPRS, Brief Psychiatric Rating Scale; DAS, Disability Assessment Scale.

* *p* < 0.05

** *p* < 0.01

*** *p* < 0.001.

### 3.4 Efficacy of psychoeducational family intervention on family functioning at one‐year

We observed a reduction in the levels of family objective (*p *< 0.01) and subjective burden (*p *< 0.001) and a significant improvement in the levels of perceived professional support (*p *< 0.001) after one year from the end of the intervention in the experimental group. There were no significant differences in the control group (Table 3).

We also observed an improvement of coping strategies of treated relatives. In particular, family members more frequently adopted problem‐oriented coping strategies, such as positive communication with the patient (*p *< 0.01) and seek for information (*p *< 0.05), and less frequently used emotion‐focused strategies, such as collusion (*p *< 0.0001), resignation (*p *< 0.001) and avoidance (*p *< 0.01) (Table 4). In the control group, we found an increase of coercion (*p *< 0.001), and a reduction of patients’ involvement in social activities (*p*<.05) and of positive communication with the patients (*p *< 0.01) (Table 4).

**TABLE 4:** Efficacy of the intervention on relatives’ coping strategies (*N *= 155)

	Experimental group (*N* = 85)		Control group (*N* = 70)	
	T0 mean (sd)	T2 mean (sd)	T0 mean (sd)	T2 mean (sd)
Collusion	2.1 (0.4)	1.9 (0.3)**	2.1 (0.4)	2.1 (0.4)
Patients’ involvement in social activities	3.0 (0.7)	3.0 (0.7)	3.1 (0.7)	2.8 (0.7)*
Resignation	2.1 (0.9)	1.6 (0.7)***	1.9 (1.0)	2.0 (1.0)
Avoidance	1.4 (0.7)	1.2 (0.4)**	1.3 (0.6)	1.4 (0.7)
Coercion	2.0 (0.6)	1.9 (0.4)	2.0 (0.6)	2.3 (0.5)***
Relatives’ maintenance of social interests	2.7 (0.7)	3.0 (0.7)***	2.5 (0.8)	2.4 (0.8)
Positive communication	3.1 (0.6)	3.3 (0.5)**	3.1 (0.6)	2.9 (0.6)**
Seek for information	2.4 (1.0)	2.7 (0.9)**	2.3 (0.9)	1.9 (0.9)
Use of alcohol and drugs	1.0 (0.3)	1.1 (0.4)	1.2 (0.6)	1.1 (0.5)
Talking with friends about patient's	2.1 (0.9)	1.9 (0.9)	1.9 (1.0)	1.9 (0.9)

Note

Abbreviation: sd, standard deviation.

* *p* < 0.05

** *p* < 0.01

*** *p* < 0.001.

### 3.5 Linear regression models

The efficacy of the intervention on patients’ symptoms and global functioning has been confirmed by the linear regression models, which explained 31.3% of the total variance for DAS global score, 55.2% of variance for the BPRS depressive/anxiety subscale, 45.2% of variance for the negative subscale and 23.5% of variance for the positive symptom subscale.

At one year, patients receiving the experimental intervention had a better social functioning and lower depressive/anxiety, negative and positive symptoms (Table 5).

**TABLE 5:** Linear regression models to test the efficacy of the intervention on patients’ global functioning and levels of psychopathology after one year

	DAS	Depressive Anxiety symptoms	Negative symptoms	Positive symptoms
Number of subjects included in the analysis	123	123	123	123
F (df)	6.49 (10)	11.05 (10)	14.824 (10)	4.52 (10)
P	<0.0001	<0.0001	<0.000	<0.000
Adjusted R square	0.317	0.460	0.539	0.230
Constant	1.15 (0.33 to 3.071)	0.52 (−0.10 to 1.13)	0.45 (0.04 to 0.85)	0.53 (0.06 to 1.00)
	B (95% Cis)	B (95% CIs)	B (95% CIs)	B (95% CIs)
Experimental treatment	−0.35 (−0.65 to −0.05)*	−0.24 (−0.45 to −0.03)*	−0.14 (−0.27 to 0.00)*	−0.23 (−0.37 to −0.68)**
Patient's gender, female	0.25 (−0.05 to 0.56)	0.19 (−0.02 to 0.41)	0.6 (−0.07 to 0.21)	−0.15 (−0.01 to 0.31)
Patient's age	−0.08 (−0.06 to 0.02)	0.03 (−0.01 to 0.01)	0.9 (−0.00 to 0.01)	−0.01 (−0.03 to 0.01)
Patient's level of education	−0.03 (−0.07 to 0.01)	−0.02 (−0.05 to 0.06)	−0.02 (−0.04 to 0.00)*	−0.09 (−0.34 to 0.01)
Employment, yes	−0.01 (−0.30 to 0.33)	−0.14 (−0.36 to 0.08)	−0.02 (−0.16 to 0.12)	−0.01 (−0.16 to 0.18)
Number of key‐relatives	0.12 (−0.9 to 0.33)	0.10 (−0.5 to.25)	0.2 (−0.07 to 0.12)	0.03 (−0.09 to 0.14)
Month on caseload of mental health center	0.01 (−0.00 to 0.00)	0.00 (−0.00 to 0.00)	0.00 (0.00 to 0.00)	0.00 (−0.00 to 0.00)
BPRS‐positive symptoms at baseline	0.25 (−0.41 to 0.90)*	0.18 (−0.28 to 0.62)	0.01 (−0.29 to 0.31)	0.67 (0.32 to 1.01)****
BPRS‐negative symptoms at baseline	0.47 (0.10 to 0.84)*	0.11 (−0.14 to 0.37)	0.59 (0.42 to 0.76)****	−0.02 (−.22 to 0.18)
BPRS‐depression/anxiety symptoms at baseline	0.30 (−0.79 to 0.53)***	0.49 (0.34 to 0.66)****	0.13 (0.02 to 0.23)*	0.35 (0.09 to 0.16)
BPRS‐manic/hostility symptoms at baseline	−0.02 (−.48 to 0.45)	−0.06 (−0.38 to 0.25)	−0.31 (0.24 to 0.17)	−0.17 (−0.41 to 0.07)

Abbreviations: B, Beta Coefficient; BPRS, Brief Psychiatric Rating Scale; CIs, Confidence Intervals; DAS, Disability Assessment Scale; df, degree of freedom.

* *p* < 0.05

** *p* < 0. 01

*** *p* < 0.001

**** *p* < 0.0001.

### 3.6 Five‐year efficacy of the experimental intervention

The efficacy of the experimental intervention was maintained after five years from the end of the intervention. In particular, we found significant differences between the experimental and the control groups in terms of patients’ relapses (28.3% in the experimental group vs. 44.4% in the control group, *p *< 0.05), number of hospitalizations (16.7% in the experimental group vs. 30.2% in the control group, *p *< 0.01) and total number of suicide attempts (0% in the experimental group vs. 9.5% in the control group, *p *< 0.01) (Table 6). The positive effect of the experimental intervention on the likelihood of having a relapse or hospitalization in the five years following the end of the intervention was confirmed by the linear and logistic regression models (Table 7). Regression models were not applicable for suicide attempts, since there were any in the experimental group.

**TABLE 6:** Five‐year efficacy of the intervention

	Experimental group (*N* = 49)	Control group (*N* = 50)
Relapse *N* (%)	17 (28.3)	28 (44.4)*
Number of relapse M ± SD	2.5 ± 2.2	2.4 ± 1.7
Hospitalization *N* (%)	10 (16.7)	19 (30.2)**
Days of hospitalization M ± SD	32.2 ± 37.7	40.8 ± 35.2
Suicide's attempt *N* (%)	0	6 (9.5)**

Note

Abbreviations: M, Mean; SD, Standard Deviation.

* *p* < 0.05

** *p* < 0.01.

**TABLE 7:** Logistic regression models to test the efficacy of the intervention on patients’ relapses and hospitalizations after five years from the end of the intervention

	Relapses	Hospitalizations
Nagelkerke R square	0.394	0.317
P	<0.0001	<0.0001
constant	7.09	5.84
	B (95% CIs)	B (95% CIs)
Experimental treatment	−1.20 (0.12 to 9.21)*	−0.99 (0.95 to 7.64)*
Patient's gender, female	−0.39 (0.25 to 1.88)	−0.97 (0.12 to 1.17)
Patient's age	0.90 (0.87 to 0.97)	−0.07 (−0.88 to −0.99)*
Patient's level of education	−.06 (−0.07 to 0.66)	−0.15 (0.73 to 1.01)
Patient's employment	0.93 (0.87 to 7.38)	−0.52 (0.19 to 1.89)
Month on caseload of mental health center	0.00 (1.00 to 1.02)*	0.00 (1.00 to 1.02)
Number of key‐relatives	0.07 (0.46 to 2.50)	−0.26 (0.28 to 2.11)
BPRS‐positive symptoms	−1.80 (0.16 to 1.66)	−2.26 (0.01 to 1.4)
BPRS‐negative symptoms	−1.28 (0.07 to 1.11)	−1.24 (0.05 to 1.62)
BPRS‐depression/anxiety symptoms	0.70 (0.92 to 2.90)	0.57 (0.73 to 3.78)
BPRS‐manic/hostility symptoms	−0.66 (0.09 to 8.90)	0.63 (0.37 to 9.28)

Abbreviations: B, Beta Coefficient; BPRS, Brief Psychiatric Rating Scale; CIs, Confidence Interval.

* *p* < 0.05.

## 4 DISCUSSION

To our knowledge, this is the first study exploring the efficacy of psychoeducational family intervention in patients with bipolar I disorder in 2 follow‐up periods, at 1 and 5 years after the end of the intervention. Our sample can be considered representative of the Italian population of patients with BDI, given the random selection of participating mental health centres, after stratification for geographic area and population density.

The efficacy of PFI in bipolar disorder has been investigated mainly in short to medium‐term follow‐up studies (usually no more than one year), while only two studies have tested its effectiveness after four^27^ and 5 years,^28^, ^29^ but in both studies the intervention was provided in group format.

**FIGURE 1:**
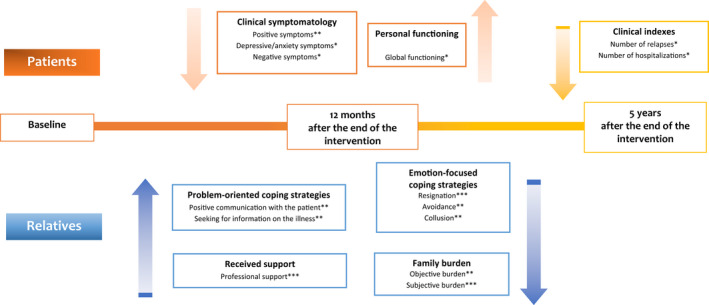
Effect of the experimental intervention on patients’ and relatives’ outcome measures at 1 and 5 years. *p < 0.05; **p < 0.01; ***p < 0.001

Although the efficacy of the experimental intervention immediately after the treatment had been already confirmed in our previous analyses,^6^, ^8^ we found that the intervention significantly improved patients’ positive, negative and depressive/anxiety symptoms, and their global functioning, at the medium‐term follow‐up (Figure 1). These results, which are largely overlapping with those found immediately after the end of the intervention, highlight that the positive effects of the intervention on patients’ symptoms and global functioning are sustained over time, and that this intervention may be useful in stabilizing patients from an illness characterized by mood instability and emotional overreactivity. This medium‐term effect may be due to increased adherence to medications,^30^ a better recognition of early warning signs (with consequent reduction of clinical relapses), improvement of patients’ and relatives’ problem‐solving skills, or improvement of the family functioning, which is a well‐known risk factor for relapses.

One important finding of our study is the significant improvement of patients’ psychosocial functioning after one year from the end of the intervention. While in the long‐term the efficacy of PFI has been mainly investigated in terms of relapse prevention and symptom reduction,^2^, ^12^ its effects on patients’ psychosocial functioning has been less frequently considered as an outcome measure. Since BD is usually associated with a significant impairment in patients’ autonomy, social, working and family functioning, this finding may be extremely important in clinical practice^31^ as it suggests the need for an integrated pharmacological and psychosocial approach in BD.

Moreover, the improvement of relatives’ coping strategies has also been considered among the outcome measures of efficacy of the experimental intervention (Figure 1). Despite the evidence clearly shows the negative impact of family instability and maladaptive coping strategies on the long‐term outcome of bipolar disorder, relatives’ coping strategies have been considered only rarely as a proxy outcome measure.^32^, ^33^ When relatives adopt positive coping strategies, patients feel less stigmatized and stressed, and report a reduced rate of relapses and hospitalizations. In our study, we found a significant improvement of relatives’ coping strategies in the group receiving PFI, and increasing levels of maladaptive coping strategies (including coercion) in non‐treated relatives. The high levels of maladaptive coping strategies in the control group may explain the worse clinical outcome found at five years in this group, confirming the important role of family members in the recovery from bipolar disorder.^34^, ^35^

We also found that PFI reduced family burden one year after the end of the intervention. This finding, which is in line with other studies on the impact of PFI on family burden in bipolar disorder^36^ and schizophrenia,^37^ may contribute to the better patients’ clinical outcome at 5 years, confirming the need for these patients to live in stable emotional environments at family and social levels.

One of the mains strengths of the study is the evaluation of the efficacy of PFI at 5 years. In particular, we observed a significant reduction in the number of relapses, hospitalizations and suicide attempts in treated patients (Figure 1). This finding confirms that psychoeducation should be considered an integral part of the “disease‐management training” of bipolar I disorder^28^, ^29^ and that it should be routinely provided to these patients according to the recovery‐oriented model of mental disorders. In order to improve its availability in routine settings, virtual settings should be explored in terms of efficacy and feasibility.^38^, ^39^, ^40^, ^41^, ^42^

Although not significant, we also found a reduction in the total length of hospitalization in treated patients compared to patients from the control group (32.2 ± 37.7 vs. 40.8 ± 25.2 days), confirming that providing PFI may be associated with a significant reduction of the costs of bipolar disorder.

For the whole duration of the study, all enrolled patients took regularly the prescribed medications according to NICE Guidelines. Patients who discontinued the pharmacological treatment have been excluded from the analyses. Given the small sample size at 5 years, the mediating effect of the different mood stabilizers on PFI has not been assessed. Further studies may help to verify whether the efficacy of family psychoeducation on the course of bipolar disorder may be at least partially mediated by mood stabilizers such as lithium.

The study has some limitations, such as the lack of an active control group and the use of different outcome measures for medium‐ and long‐term assessments. However, the study was conceived as a “real‐world” study and therefore it was not possible to include another active intervention as comparator besides TAU, nor to include an in‐depth assessment after five years. We decided to select a limited number of hard clinical indicators in order to collect information on as many patients as possible minimizing the drop‐out rate. The reduced number of patients who have been reassessed at 5 years (99 at T3 vs 137 at T0) is another possible limitation of the study. However, we found no statistically significant differences in patients’ and relatives’ socio‐demographic and clinical characteristics between T0 and T3 assessments. Finally, one more possible limitation is the use of the BPRS to assess affective symptoms rather than more specific instruments for bipolar disorder. This choice was due to the fact that the BPRS is a well‐known instrument frequently used in ordinary psychiatric settings, and it can be easily used by mental health professionals with different background and after a brief training.^43^

The present study gave us the opportunity to assess the efficacy of a single‐family psychoeducational intervention in real‐world settings over a medium‐ and long‐term period. According to our findings, the provision of psychoeducational family intervention is associated with several positive outcomes for both patients and relatives. In particular, at one year, the intervention was particularly effective in improving patient's clinical status and psychosocial status, as well as relatives’ coping strategies and perceived professional help, and in reducing family objective and subjective burden. Moreover, for the first time our study showed that a six‐month single‐family psychoeducational intervention is associated with a five‐year improvement of several clinical hard indicators, including number of patients’ relapses, hospitalizations and suicide attempts, suggesting a possible impact of the intervention in reducing the costs of illness. Moreover, the intervention was well received by patients and relatives, as demonstrated by the high retention rate. According to our results, it is possible to conclude that the provision of a psychoeducational family intervention in real‐world setting is associated with significant and long‐lasting positive effects on patients’ and relatives’ mental health and well‐being. Strategies should be implemented worldwide in order to provide this intervention in routine settings for patients with bipolar I disorder and their relatives.

## ACKNOWLEDGEMENTS

Open Access Funding provided by Universita degli Studi della Campania Luigi Vanvitelli within the CRUI‐CARE Agreement.

## CONFLICT OF INTEREST

Authors declare to have no conflict of interests.

## Data Availability

The data that support the findings of this study are available on request from the corresponding author. The data are not publicly available due to privacy or ethical restrictions.
